# Hydrolyzed Feather Meal in Diet of Yellow Catfish (*Pelteobagrus fulvidraco*): Effects on Growth Performance, Flesh Quality, Skin Color, and Intestinal Flora

**DOI:** 10.1155/anu/7200771

**Published:** 2025-08-31

**Authors:** Shuang Zheng, Tongqiang Wu, Lei Zhong, Peng Li, Yi Hu, Junzhi Zhang

**Affiliations:** ^1^College of Fisheries, Hunan Agricultural University, Changsha 410128, China; ^2^Department of Animal Nutrition, North American Renderers Association, Alexandria, Virginia 22314, USA

**Keywords:** fish meal, flesh quality, growth, hydrolyzed feather meal, intestinal flora, *Pelteobagrus fulvidraco*

## Abstract

An 8-week feeding trial was conducted to assess the effects of hydrolyzed feather meal (HFM) as a fish meal replacement on the growth performance, flesh quality, skin color, and intestinal microbiota of yellow catfish (*Pelteobagrus fulvidraco*). Five isonitrogen (44% crude protein) and isolipidic (8.5% crude lipid) diets were formulated with varying levels of HFM at 0% (FM, control), 2.05% (HFM2), 4.10% (HFM4), 6.15% (HFM6), and 8.20% (HFM8), corresponding to fish meal replacement of 0%, 8.33%, 16.67%, 25%, and 33.33%, respectively. Results indicated that the growth performance declined significantly as HFM inclusion increased. Based on the results of weight gain rate (WGR) and specific growth rate (SGR), the maximal replacement levels of fish meal with HFM for yellow catfish should be 16.67%. Intestinal enzyme activities, including trypsin, lipase, amylase, and Na^+^–K^+^–ATPase as well as villus height, muscular thickness, and goblet cells number were significantly enhanced in HFM groups. Fish meal replacement with HFM remarkably reduced serum immune indicators acid phosphatase, immunoglobulin M, Complement 3, and Complement 4 levels and significantly increased serum aspartate aminotransferase, total triglycerides, and cholesterol levels, indicating compromised immune function and liver health. The content of collagen and flavor-enhancing amino acids (glutamic acid [Glu], glycine [Gly], and alanine [Ala]), as well as muscle hardness were distinctly boosted, demonstrated an elevated flesh texture led by dietary HFM inclusion. The abnormal skin coloration induced by pigmentary disorders was observed in high HFM inclusion groups, the black pigmentation on dorsal and yellow pigmentation on the abdomen exhibited a gradual reduction in intensity. The study found that replacing up to 16.67% of fish meal with HFM in yellow catfish diets maintained growth performance and improved meat quality. However, high HFM levels damaged serum immune system and caused liver dysfunction, dyslipidemia, pigmentary disorders, and reshaped intestinal microbial structure.

## 1. Introduction

As the most optimal protein source for fish, fish meal possesses not only a high protein content but also a balanced profile of amino acids, along with abundant fatty acids and vitamins. Additionally, it exhibits favorable characteristics of good palatability, high digestibility, and feeding attraction [1]. However, the paradoxical relationship between the burgeoning demand and the limited production of fish meal has resulted in an oversupply, leading to escalating prices. This situation has greatly elevated the cost of breeding and aggravated the burden on farmers. To address this challenge, numerous studies have been reported on alternative protein sources to fish meal, such as animal protein sources [2], plant protein sources [3], and yeast [4]. Animal protein is typically derived from by-products of aquatic products, livestock, and poultry processing. Compared with plant protein sources, it boasts advantages of high utilization rate, low antinutritional factors, plentiful minerals, vitamins, and amino acids [5]. Nevertheless, the imbalance of amino acid content and high fat saturation in by-products of livestock and poultry processing such as feather meal adversely affected the palatability, which always considered as an important reason explaining the decline in fish growth when an excessive animal protein were added [6].

Feather meal mainly refers to the product obtained from feathers removed during the slaughtering of poultry after appropriate technical processing. However, the keratin in feather meal exhibits remarkable structural stability due to its high degree of cross-linking through disulfide bonds and hydrogen bonding networks. This unique configuration renders it highly resistant to proteolytic digestion by conventional hydrolytic enzymes. Native feather keratin demonstrates remarkably low digestibility when treated with pepsin or trypsin [7]. Consequently, high-pressure heating, hydrolysis, and puffing are commonly used to breakdown the keratin structure in feathers, making feather components more soluble before feather meal utilization [8]. This process converts keratin into soluble proteins, facilitating digestion and absorption. Research on hydrolyzed feather meal (HFM) as a source of protein in aquatic animals has been limited up to this point. Prior research has demonstrated that substituting 33% of fish meal with HFM in the diet of Nile tilapia (*Oreochromis niloticus*; control diet comprising 22% fish meal) [9] and 76% of fish meal in the diet of seabass (*Dicentrarchus labrax*; control diet comprising 29% fish meal) [10] did not adversely impact the growth and health of fish. However, complete fish meal replacement with HFM resulted in a severe growth inhibition of gilthead seabream (*Sparus aurata*), which was attributed to the imbalance of amino acids in HFM, as well as the poor palatability of the meal [11, 12]. Despite these studies, the impact of HFM on meat quality and body coloration, key market traits for species like yellow catfish, remains unexplored. Addressing this gap is essential for evaluating holistic suitability of feather meal in aquafeeds.

Fish flesh quality and body color fluctuate market price and purchasing decisions to a large extent. Yellow catfish possessing the properties of firm texture, fresh taste, and bright skin color are preferred by consumers. Among meat quality attributes, flesh texture, a sensory characteristic, influences the meat's mouthfeel during mastication and serves as an indicator of freshness [13, 14]. Firmness, adhesiveness, cohesiveness, springiness, gumminess, tenderness, and other qualities can all be used to gauge the texture of flesh. There are numerous internal and external elements that affect the textural characteristics of muscle, such as species, age, sex, genotypes, diet, and environmental circumstances [15]. Skin color is a universal clue to the health of fish and is considered an important economic characteristic. Melanin, carotenoids, purines, and pteridine are among the pigments that mostly influence skin color. Fish skin color is easily impacted by diet, environmental stresses, and water pollutants in artificial breeding environments. Intestinal microorganisms can help host to digest food and regulate the immune system and bacterial colonization. By regulating fish intestinal microbes, changing metabolic processes, and reducing the number of significant symbiotic species, diet composition can impact host intestinal homeostasis. To data, evaluation studies on HFM as a potential protein source from perspective of flesh quality, skin coloration, and enteric microorganism have not been reported.

Yellow catfish, *Pelteobagrus fulvidraco*, one of the important freshwater economic fish species in China, is a carnivorous fish with a high fish meal requirement, generally ranging from 28%–35% [16]. Fish meal accounts for a large proportion of feed cost which seriously hampers the development of yellow catfish industry. Searching for an alternative protein for fish meal has become pivotal. In order to provide a theoretical foundation for the use of HFM in fish feeds and lower the total cost of aquaculture, this study intends to examine the effects of replacing fish meal with HFM on the growth, flesh quality and skin color, and intestinal flora of yellow catfish.

## 2. Materials and Methods

### 2.1. Experimental Diets

The primary protein sources in the experimental diets were fish meal, soybean meal, cottonseed meal, and rapeseed meal, whereas the lipid source was soybean oil. Five isoprotein (44% crude protein) and isolipidic (8.5% crude lipid) experimental diets were developed based on the nutritional needs of yellow catfish. The control diet (FM) was set to contain 30% fish meal, and HFM was used to replace 8.33%, 16.67%, 25%, and 33.33% of the fish meal in the control diet, respectively (designed as HFM2, HFM4, HFM6, and HFM8). The experimental formula is shown in Table 1 and the feed preparation methods refer to the previous steps [17]. The diets were subjected to a chemical analysis using accepted techniques [18]. The amino acid profile of the diets and muscle were analyzed by high-speed amino acid analyzer (L-8900, Hitachi High-Tech Science, Tokyo, Japan) and determined following the methods described by Cai et al. [19]. Amino acids compositions and content of HFM and fish meal are shown in Table 2, and amino acids compositions and content of experimental feed are shown in Table 3.

### 2.2. Feeding Experimental Procedure

The Chetianjiang reservoir served as the site of the feeding experiment. Juvenile yellow catfish were bought from a commercial farm in Xiangyin, Hunan, and raised for a week in floating cages (5.0 m × 5.0 m × 5.0 m) to get used to the experimental setup. Yellow catfish were given the control diet during this time. All young fish were fasted for 24 h before the feeding experiment, after which uniformly proportioned individuals (10.50 g) were chosen for weighing and allocated at random to 15 cages (2.0 m × 2.0 m × 2.0 m). Groups of different diets were randomly divided into three repeating groups with 50 fish per cage. The feeding and management methods during the experiment refer to the previous reports [17].

### 2.3. Sample Collection and Analysis

#### 2.3.1. Growth Performance

Fish were given a 24-h fast at the conclusion of the feeding session. To calculate the survival rate (SR), weight gain rate (WGR), specific growth rate (SGR), and feed conversion rate (FCR), each fish in the cage was sedated with 100 mg/L MS-222 (Shanghai Reagent Corp., Shanghai, China). The fish were then collectively weighed and counted. In order to calculate the hepatosomatic index (HSI), relative intestinal length (RIL), and intestosomatic index (ISI), the entire liver and intestine of six fish per cage were dissected and their weight and length were noted.

#### 2.3.2. Intestinal Digestive Enzymes and Intestinal Morphology

In order to assess the digestive enzyme activities (amylase, lipase, trypsin, Na^+^–K^+^–ATPase, and AKP) using commercial kits (Nanjing Jiancheng Biotechnic Institute, Nanjing, China), the intestinal tracts of six fish per cage were separated, cleaned with PBS, and kept at −20°C. Three fish's anterior intestine were randomly selected from each cage, and tissue slices were made using the procedure by Yang et al. [20]. A light microscope with a camera and the CellSen standard software was used to take the pictures. From each slide, 10 villi were chosen at random, and their lengths from tip to base were measured. Additionally, muscle thickness and goblet cells number were measured and examined.

#### 2.3.3. Intestinal Flora

Intestinal contents from the posterior intestine of yellow catfish in each group were collected 2–4 h after feeding into 1.5 mL nuclease-free tubes (three fish per cage), rapidly frozen in liquid nitrogen, and stored at −80°C. The intestinal microbiota analysis was conducted following established protocols from our prior study, with specific methodological details provided in [21].

#### 2.3.4. Serum Parameters

A 1 mL syringe was used to draw blood from the caudal vein of six fish per cage. The blood was then collected in a 10 mL centrifuge tube, allowed to clot at 4°C for 6 h, and then, centrifuged at 3500 r/min for 10 min at 4°C. Before determining the serum characteristics (AST, ALT, TC, glutamic acid [GLU], TP, ACP, AKP, IgM, C3, and C4) using commercial kits (Nanjing Jiancheng Biotechnic Institute, Nanjing, China), the obtained serum was kept at −80°C as per the manufacturer's instructions.

#### 2.3.5. Skin Pigments

PBS was used to wash the yellow catfish's surface skin. Skin from the abdomen and dorsum in the same place was removed, collected, and preserved at −80°C. Assays for tyrosinase activity in dorsum skin were conducted in accordance with earlier reports [22]. The amounts of lutein and carotene in the skin of the abdomen were measured using the techniques outlined by Liu et al. [23] and Cejas et al. [24].

#### 2.3.6. Water Holding Capacity of Muscle

Cooking loss of muscle was determined using the method described by Wongthahan and Thawornchinsombut [25] with a slight modification. Centrifugal loss of muscle was measured by the method as described by Sánchez-Alonso et al. [26].

#### 2.3.7. Texture Analysis of Muscle (Flesh)

A texture analyzer (Tatouch, Shanghai, China) was used to examine the texture of the dorsal muscle (without skin). The 1.0 cm × 1.0 cm × 1.0 cm muscle was compressed twice at a 30% compression ratio each time, with a 5 s measurement interval and a constant speed of 1 mm/s. According to Hixson [27], chewiness, hardness, and springiness were assessed.

### 2.4. Calculation and Statistical Analysis

Data were analyzed using SPSS 19.0 software (Chicago, IL, USA). Homogeneity and normality of variances was tested using the Leven's test. Significant differences were evaluated by one-way analysis of variance (ANOVA) followed by a post hoc Tukey test. *p* < 0.05 was considered significant. Results were presented as means and standard deviation (S.D.). The treatment effects were analyzed using general linear models (GLMs) in SAS software v.9.4 M3 (SAS Institute Inc., USA). Results are presented as the mean and standard error of the mean (SEM). Linear and quadratic responses to varying levels of HFM replacement were assessed using orthogonal polynomial contrasts. Statistical significance was determined at *p* < 0.05.

## 3. Results

### 3.1. Growth Performance

Increasing HFM levels linearly reduced FBW, SGR, and WGR, with HFM6 and HFM8 groups showing significantly lower values than FM group (*p* < 0.05), which showed a significantly linear relationship with the HFM replacement level (*p* < 0.05). FCR increased significantly in HFM6 and HFM8 groups compared to FM group, HSI increased markedly even at HFM2 group, showing linear and quadratic responses to HFM replacement levels (*p* < 0.05). RIL was elevated in HFM2 group compared to FM group, ISI increased significantly in HFM2, HFM4, and HFM6 groups versus FM group, exhibiting a quadratic relationship with HFM inclusion (*p* < 0.05; Table 4).

### 3.2. Intestinal Digestive and Absorptive Enzymes Activities

Amylase activity increased significantly at HFM6 and HFM8 groups (*p* < 0.05), showing a linear response to HFM replacement level (*p* < 0.05). Trypsin activity peaked in HFM4 and HFM6 groups (*p* < 0.05), but declined at HFM8 group compared to FM group, exhibiting a quadratic relationship with HFM replacement level (*p* < 0.05). AKP activity decreased linearly (*p* < 0.05), whereas Na^+^–K^+^–ATPase content was significantly increased (*p* < 0.05), though neither showed a clear linear or quadratic trend with HFM replacement level (Table 5).

### 3.3. Intestinal Morphology

**Figure 1:**
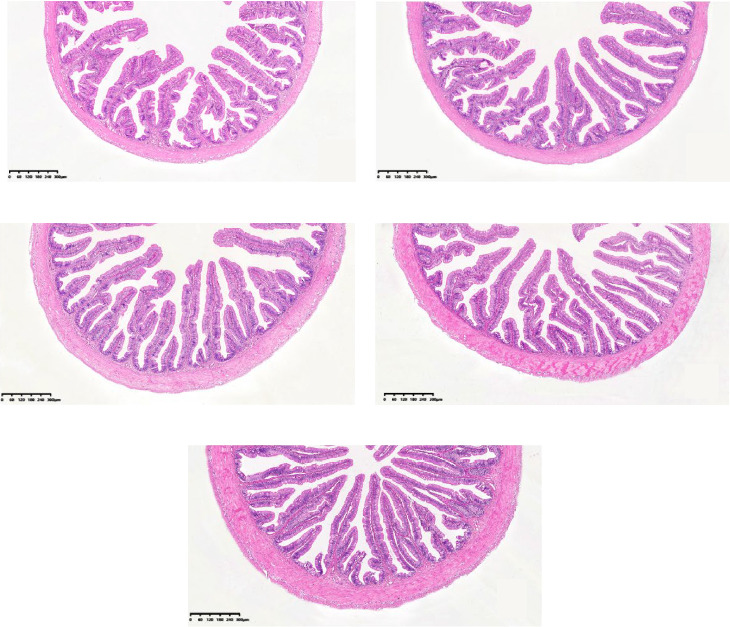
The tissue morphology of anterior intestine responding to graded levels of HFM inclusion in *Pelteobagrus fulvidraco*. FM (A), HFM2 (B), HFM4 (C), HFM6 (D), and HFM8 (E).

In the anterior intestine, HFM replacement significantly increased villus height, muscle layer thickness, and goblet cells number (*p* < 0.05). All parameters showed a significant linear response to HFM replacement level (*p* < 0.05; Table 6 and Figure 1).

### 3.4. Intestinal Microbiota Analysis

#### 3.4.1. Analysis of the Species Composition of Intestinal Microbiota

**Figure 2:**
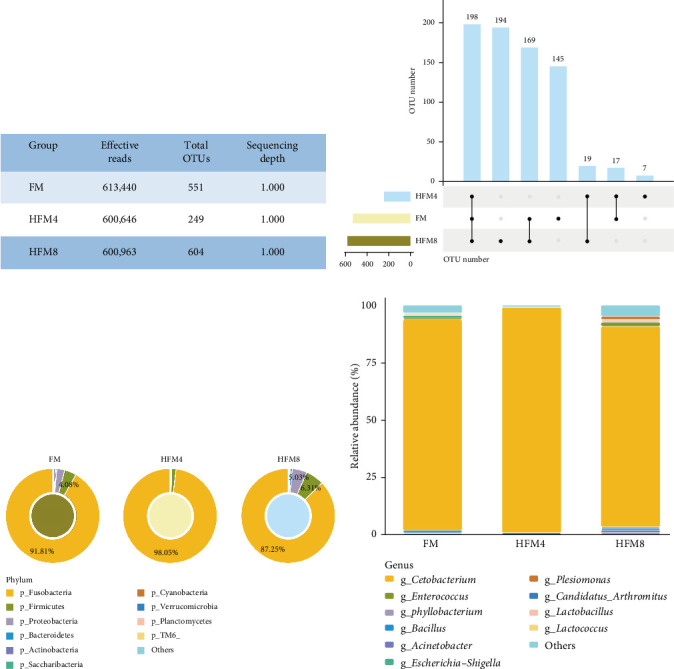
Composition of intestinal microbiota between Group FM, HFM4, and HFM8. (A) Analysis of high throughput sequencing results for each sample; (B) statistics on the number of shared and unique ASVs between groups; (C) composition of intestinal microbiota at the phylum level; (D) composition of intestinal microbiota at the genus level.

16S rRNA sequencing of intestinal microbiota yielded 1,815,049 high-quality sequences from 15 samples, clustered into 1404 operational taxonomic units (OTUs; 97% similarity). OTU counts were 551 (FM), 249 (HFM4), and 604 (HFM8). At the phylum level, dominant bacteria included Fusobacteria, Firmicutes, Proteobacteria, Cyanobacteria, and Bacteroidetes. Fusobacteria abundance peaked in HFM4 group (98.00%) and was lowest in HFM8 group (86.71%). At genus level, *Cetobacterium* dominated all groups (FM: 91.11%, HFM4: 97.96%, and HFM8: 86.64%). FM group showed secondary dominance by *Escherichia*–*Shigella* (1.13%), while the rest of the genera had relative abundances of less than 1%. In addition to *Cetobacterium*, the relative abundances of the other genera in group HFM4 were less than 1% and relative abundances of more than 1% in HFM8 were *Enterococcus* (1.63%), *Plesiomonas* (1.33%) and *Acinetobacter* (1.13%; Figure 2).

#### 3.4.2. Analysis of Microbial Diversity

**Figure 3:**
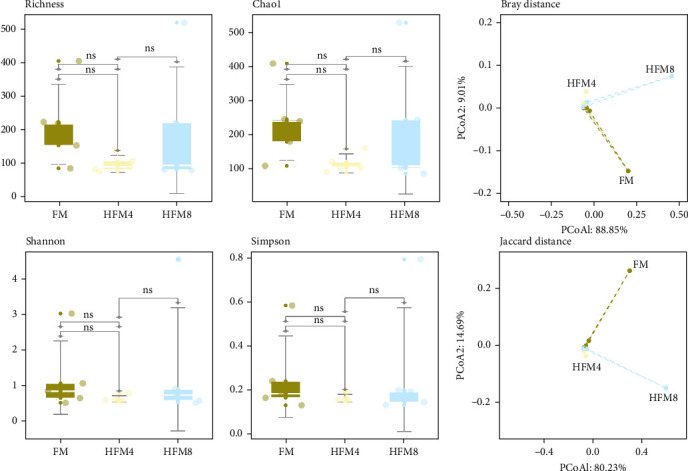
Impact of microbiological diversity.

Alpha diversity indices showed no significant differences among groups (*p* > 0.05). PCoA based on Bray–Curtis (PCoA1 = 88.85% and PCoA2 = 9.01%) and Jaccard (PCoA1 = 80.23% and PCoA2 = 14.69%) distances revealed clear separation of microbial communities, with two-dimensional plots effectively representing both abundance and structural variation (Figure 3).

**Figure 4:**
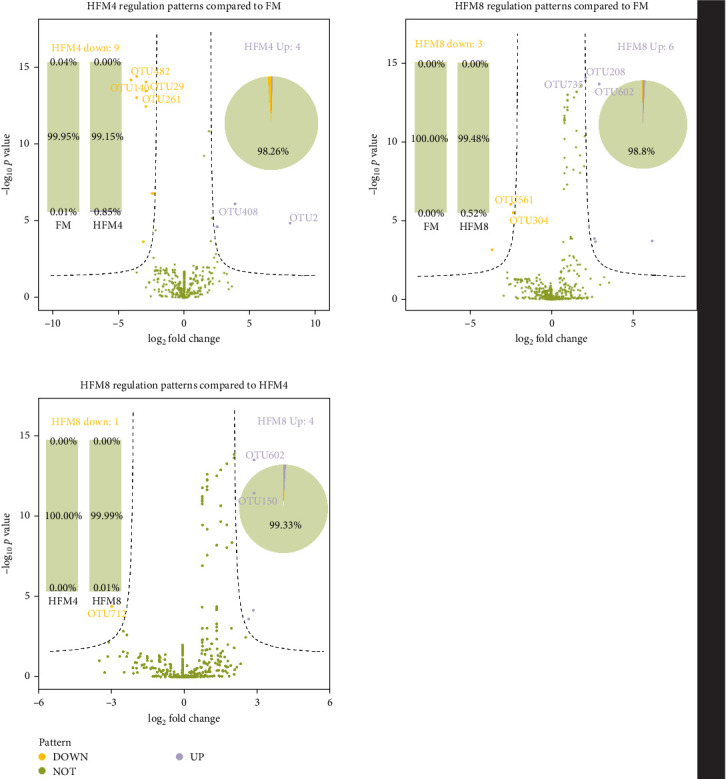
Differential markers of intestinal microbiota between Group FM, HFM4, and HFM8.

Limma analysis (fold change > 3; *p*=0.01) revealed differential OTU responses to all treatment. The number of upregulated OTUs in each group was four (HFM4) and six (HFM8) with an abundance of 0.85% (HFM4) and 0.52% (HFM8) and the number of downregulated OTUs in each group was nine (HFM4) and three (HFM8) with an abundance of 0.00% (HFM4) and 0.00% (HFM8) compare to FM group, respectively. Compared to the HFM4 group, the number of upregulated OTUs in the HFM8 group was four with an abundance of 0.01%, and the number of downregulated OTUs was one with an abundance of 0.00% (Figure 4).

### 3.5. Serum Biochemical and Immune Indexes

Dietary HFM levels linearly and quadratically affected serum AST, TG, and GLU (*p* < 0.05). The activity of serum AST and TG increased significantly with the increase of HFM replacement ratio (*p*  < 0.05), while GLU and TP were higher in HFM groups than FM group (*p*  < 0.05). HFM inclusion linearly and quadratically decreased AKP and ACP activity compare to FM group (*p*  < 0.05). HFM replacement also linearly reduced serum IgM, C3, and C4 (*p*  < 0.05; Table 7).

### 3.6. Muscle Physicochemical Index

There were no significant differences in centrifugal loss and cooking loss of muscle among the treatments (*p* > 0.05). While the ODM was significantly elevated in HFM6 group (*p* < 0.05). Muscle hardness was increased significantly when HFM substitution exceeded 8.33%, while springiness decreased with HFM inclusion (*p* < 0.05). Adhesiveness was significantly higher in HFM replacement groups (*p* < 0.05). ODM, hardness, and adhesiveness showed linear relationships with the HFM replacement level (*p* < 0.05; Table 8).

### 3.7. Free Amino Acid Content in Muscle

HFM replacement linearly increased muscle total free amino acid content. Specifically, with HFM8 group showing significantly higher levels than FM, HFM2, and HFM4 groups and showed a significantly linear relationship (*p* < 0.05). Glu, glycine (Gly), Ser, and alanine (Ala) contents also increased significantly and exhibited linear responses to HFM replacement level (*p* < 0.05; Table 9).

### 3.8. Skin Pigments

**Figure 5:**
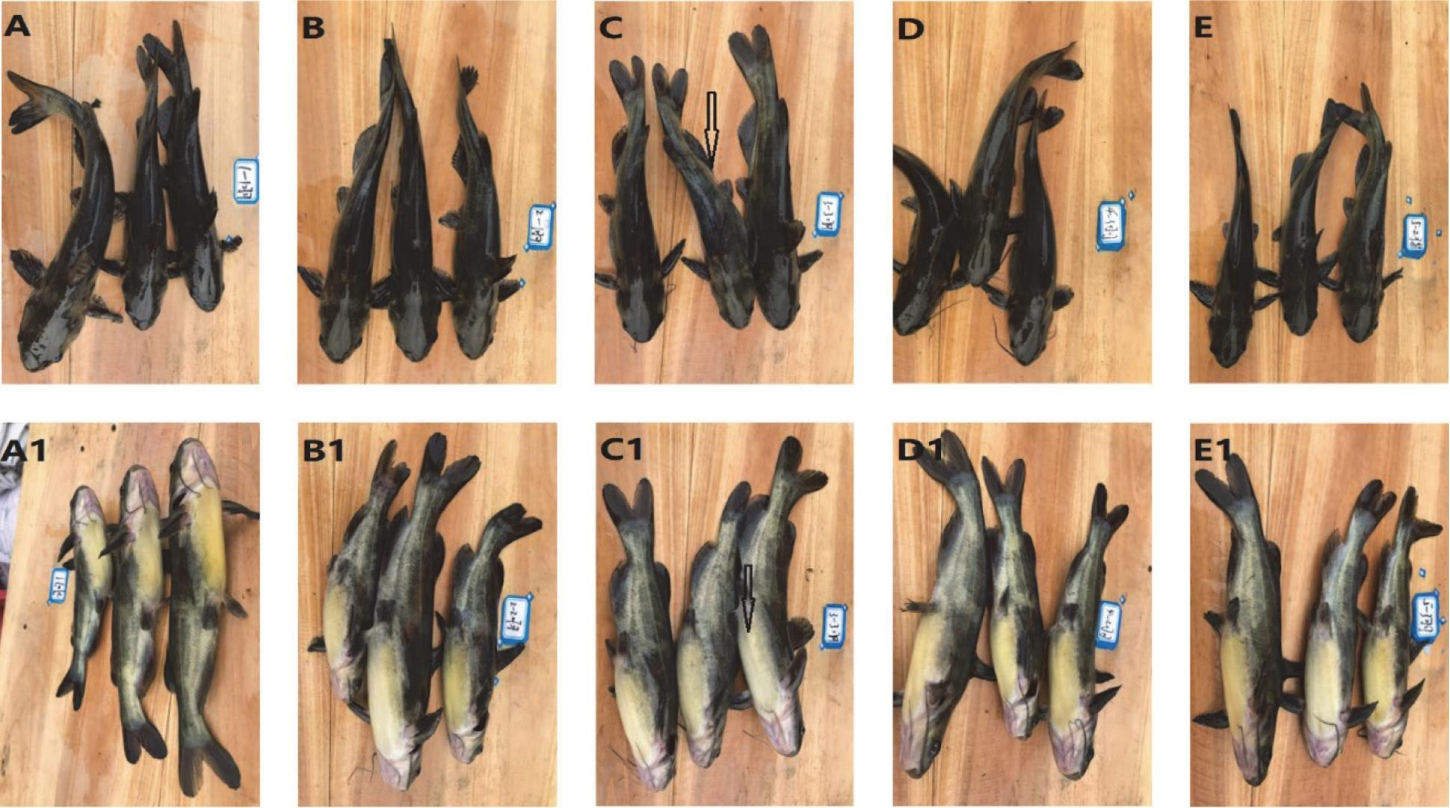
Effects of HFM instead of fish meal on body color of *Pelteobagrus fulvidraco*. FM (A, A1), HFM2 (B, B1), HFM4 (C, C1), HFM6 (D, D1) and HFM8 (E, E1).

The tyrosinase activity content in the dorsal skin, lutein, and carotene content in the ventral skin were linearly decreased to varying degrees (*p*  < 0.05; Table 10). Skin color analysis demonstrated progressive fading of both dorsal black pigmentation and abdominal yellow pigmentation with increasing HFM levels, with HFM4 group showing the most pronounced changes (Figure 5).

## 4. Discussion

To date, the most research results of HFM in aquatic animals indicated that an appropriate amount of HFM could partially replace fish meal without adverse effects on the fish physiology [28]. Paradoxically, the study of tench (*Tinca tinca* L.) found that 5% HFM inclusion in the diet reduced weight gain [29], and the same result was in carp (*Cyprinus carpio* L.) [30]. The above reports demonstrated that the utilization efficiency and tolerance of feather meal by aquatic animals is closely related to the fish species. In this study, with the increasing replacement levels, the growth performance was gradually inhibited. While only when the fish meal replacement levels reached up to 25%, WGR and SGR were significantly lower than FM group. After analysis, the content of lysine and methionine in HFM is significantly lower than that in fish meal. Supposedly, it could not meet the demand of amino acids for yellow catfish carnivorous animals, which may partially account for the depressed growth [31]. The study on channel catfish (*Ictalurus punctatus*) showed that supplementing lysine in feather meal inclusion diet enhanced its benefits [32]. Additionally, the absence of certain unidentified growth factors or tiny peptides in HFM may be the cause of the negative effects of HFM on growth performance [12, 33]. The current study's maximum replacement levels of fish meal with HFM for yellow catfish were 16.67% based on growth performance.

Digestive enzyme activity serves as an key element to determine nutrient digestion to a certain extent, and the change in the composition and level of feed could easily cause variation in the activity of digestive enzymes [34, 35]. Untreated feather meal contains a substantial number of disulfide bond molecules in its protein structure, rendering it a challenge for animals to digest and absorb after ingestion [36]. While treating with hydrolysis, enzymatic digestion and gas explosion process breaks the disulfide bond, facilitating easier digestion and utilization by aquatic animals. Even so, the apparent digestibility of processed feather meal is still far inferior to that of fish meal [37]. To acquire enough nutrient substance to meet self-requirement, therefore, yellow catfish secreted more digestive and absorptive enzyme to maximize the utilization of HFM in this experiment. In line with this, the total acid protease (pepsin) activity of gilthead seabream was improved by replacing fish meal with HFM [12]. Discrepantly, the study on the cuneate drum (*Nibea michthioides*) revealed that the addition of feather meal suppressed lipase and amylase activity in the hepatopancreas and intestines, which may be attributed to the intestinal damage caused by the nondegradable proteins contained in feather meal [38].

In addition, intestinal morphology is a critical aspect of evaluating fish intestinal health, with its structure and function being closely related to feed efficiency and animal growth [39]. Intestinal structural integrity is often measured by measuring intestinal villus height, muscle thickness, and number of goblet cells [40]. Villus height plays a crucial role in determining the surface area for intestinal digestion and absorption, with an increase in villus height contributing to the expansion of the intestinal absorptive area [41]. Goblet cells secrete mucus containing lysozyme, mucopolysaccharides, glycolipids, and digestive enzymes, which collectively form the intestinal chemical barrier. Alterations in the abundance and function of goblet cells may partially reflect changes in this chemical barrier [42]. The muscular thickness is indicative of the intestinal contraction strength and the increase in thickness enhances the forcefulness of intestinal contractions, which is conducive to digestion [43]. The anterior intestine of yellow catfish is the main site of digestion and absorption [44]. In this experiment, the villus height, muscular thickness, and goblet cells number significantly increased with the increase of the substitution ratio. Moreover, RIL and ISI were increased. This demonstrated a promoted capacity of digestion and absorption through reinforcing morphological structure of intestine. Speculatively, it is also a compensatory response.

Under normal circumstances, microbial flora symbioses with the host in specific ways such as the skin and intestine, which facilitates a variety of biological processes, such as growth, immunomodulation, disease resistance, metabolism, absorption, and digestion [45]. This experiment showed that the dominant flora in the intestines of different groups at the phylum level was the same, it is mainly composed of Fusobacteria, Firmicutes, and proteobacteria. Fusobacteria and Firmicutes are considered beneficial bacteria and have been reported in the intestinal of yellow catfish [46], grass carp (*Ctenopharyngodon idella*) [47], and zebrafish (*Barchydanio rerio var*) [48]. Statistical analysis of the flora structure and distribution of each sample at the genus level revealed that *Cetobacterium* were the dominant species in groups FM (91.11%), HFM4 (97.96%), and HFM8 (86.64%), aligning with the results of Zhang et al.'s [49] study on the structure of intestinal flora of yellow catfish. Generally, a high diversity of microbial flora implies a higher resistance and resilience to environmental stress, playing a key role in maintaining the health status of the host [50]. In this experiment, compared to the FM group, the bacterial flora abundance and diversity decreased in the HFM4 group, while the HFM8 group showed similarity to the FM group based on Shannon, Simpson, and ACE indices. Principal component analysis (PCA) of OTUs indicated a high overall similarity among the three groups, yet substantial differences in community composition, suggesting that HFM had an effect on the species and quantity of intestinal flora of yellow catfish. The replacement of fish meal with 16.67% HFM in the feed resulted in a potential inhibition of the growth of intestinal microorganisms, which may be associated with the negative digestibility of HFM. This implies that the effect of HFM on the abundance and diversity of intestinal flora may be related to the substitution level. Currently, there is limited literature on the study of HFM in the diet of yellow catfish on intestinal flora, and further research is needed to validate this conclusion.

The liver is a major regulator of lipid metabolism in the body. Liver dysfunction readily causes abnormal lipid metabolism, further disturbing serum lipid metabolite profile. Serum TG and TC levels are indicative of the body's lipid metabolism. ALT and AST are indicators of liver function in fish, elevated levels of which indicate disturbances in lipid metabolism and impaired liver function [51]. The results revealed that HFM led to a significant increase in serum AST and TG levels, demonstrating that the HFM inclusion aggravated liver function injure and potentially caused lipid metabolism disorder. Fish mainly rely on nonspecific immune defense system to clear foreign matter, with AKP and ACP activities are currently commonly used as indicators for evaluating nonspecific immunity in fish [52]. The results of this study revealed a significant decrease in serum AKP, ACP, IgM, C3, and C4 levels following the substitution of fish meal with HFM, indicating that the high proportion of substitution compromised fish immune system to a certain extent.

In order to provide premium quality and ensure consumer approval for human health, the evaluation of fillet freshness is crucial in research and development [53]. Several characteristics, including hardness, cohesiveness, springiness, chewiness, resilience, and adhesiveness as well as the internal cross-linking of connective tissue and the detachment of fibers, are used to evaluate the freshness quality of fish muscle [54]. In this study, muscle hardness was significantly increased after the HFM substitution level exceeded 8.33%, aligning with the notable increase in ODM. In texture properties, hardness, also known as firmness, is closely related to the visibility and acceptability of fish products to human. This vital index depends largely on the structure of connective tissue. The cross-linking within collagen fibers creates a tightly connected, stable network structure, enhancing both toughness and mechanical strength. Hence, collagen cross-linking had the greatest contribution to the fillet firmness. Comparisons among species reveal that fish with firmer fillets tend to have a higher collagen fibril density within connective tissues.

Amino acid content is an important basis for evaluating nutritional value, flavor quality, and breeding regulation. The yellow catfish is rich in essential amino acids that cannot be synthesized by the human body, such as lysine, methionine, and tryptophan. The content and proportion of these amino acids directly determine the biological value and nutritional balance of the protein. Free amino acids serve as precursors for protein synthesis in the body, contributing to the maintenance of normal life activities and energy supply for the development of animal larvae [55]. Additionally, free amino acids are mainly flavor substances and flavor precursors of nonvolatile taste, with different flavor characteristics categorized into sweet and umami amino acids [56, 57]. In this experiment, substitution levels exceeding 16.67% resulted in a significant increase in muscle total free amino acid content. The notable increase in total free amino acid was primarily attributed to elevated levels of Glu, Gly, and Ala in the current study. The free amino acid Gly and Ala have a sweet taste, while Glu is closely related to the umami taste of aquatic products [58]. This indicated that HFM inclusion enhanced the flesh flavor.

Skin coloration acts as a more general cue to fish health and is considered as a vital economic trait immensely fluctuating the market acceptability and price. In the breeding of yellow catfish, nutritional components have a significant impact on the synthesis of pigments in the back and abdomen, mainly achieved by regulating the intake of carotenoids and the synthesis of melanin. Among pigments, carotenoids, including carotene and lutein, cannot be synthesized endogenously and only provided exogenously by the feeds [59]. Yellow and red body colors were typically determined by carotene and lutein [60]. Black coloration primarily dependent on the content and distribution of melanocytes. Fish can synthesize melanin, melanin darkens fish skin, with tyrosinase serving as the rate-limiting enzyme in the regulation of melanogenesis [61]. Methionine deficiency will reduce the activity of tyrosinase, affect the conversion of tyrosine to L-DOPA and dopaquinone, and thereby reduce melanin production [62]. The yellow catfish is characterized by a dark brown dorsum and a light yellow abdomen. In the current study, the inclusion of HFM decreased tyrosinase activity in the dorsal skin, indicating lower melanin formation. This might be due to the fact that the methionine content in HFM is lower than that in fish meal. Additionally, the content of lutein and carotene was reduced following HFM inclusion, consistent with the observed variation in skin color. These results indicated that HFM inclusion caused the pigmentary disorders and abnomal skin coloration. The absorption, utilization, and synthesis of pigments are the complex regulatory processes and disruption of any key step would induce abnormal skin coloration. It has been reported that oxidative stress played a notable role in melanogenesis [63]. Regrettably, the oxidative stress status was not be tested in the current study. The regulation mechanism underlying melanin disorders induced by HFM inclusion need to further be explored. The absorption of dietary carotenoids primarily takes place in the intestine [64], the reduced lutein and carotene deposition could be attributed to the lower digestibility of HFM inclusion diet, resulting in insufficient intake of lutein and carotene.

## 5. Conclusion

In this study, the growth performance was gradually depressed with the increasing replacement levels. Additionally, the capacity of digestion and absorption were elevated followed HFM added. Unexpectedly, HFM treatment increased the content of flavor amino acids and improved flesh texture, which are closely associated with meat quality. However, high HFM levels damaged serum immune system and caused liver dysfunction, dyslipidemia, pigmentary disorders, and reshaped intestinal microbial structure. Based on the comprehensive results of growth, immunity, digestion, and muscle quality, the recommended substitution level in practical application is 16.66%. This study provided a theoretical basis for the application of HFM in fish feeds.

## Figures and Tables

**Table 1:** Formula and ingredients content of the experimental feed (%, dry matter).

Ingredients	FM	HFM2	HFM4	HFM6	HFM8
Fish meal	30	27.5	25	22.5	20
Hydrolyzed feather meal^a^	0	2.05	4.10	6.15	8.20
Soybean meal	18	18	18	18	18
Cottonseed meal	4	4	4	4	4
Rapeseed meal	4	4	4	4	4
Beer yeast	6	6	6	6	6
Corn gluten powder	5	5	5	5	5
Strong flour	25	25	25	25	25
Calcium dihydrogen phosphate	1.5	1.5	1.5	1.5	1.5
Soybean oil	3.9	4.1	4.3	4.5	4.7
Vitamin and mineral premix^b^	1.2	1.2	1.2	1.2	1.2
Choline chloride	0.5	0.5	0.5	0.5	0.5
Antioxidant	0.01	0.01	0.01	0.01	0.01
Mold inhibitor	0.03	0.03	0.03	0.03	0.03
Wheat middlings	0.86	1.11	1.36	1.61	1.86
Total	100	100	100	100	100
Proximate analysis^c^					
Crude protein	44.52	44.38	44.14	44.61	43.96
Crude lipid	8.51	8.63	8.57	8.39	8.70
Crude ash	11.21	11.16	11.34	11.19	11.28

^a^The hydrolyzed feather was provided by American Animal Protein and Oil Extraction Association (Washington, America).

^b^The premix provided by MGOTer Bio-Tech Co.Ltd (Qingdao, Shandong, China) and provided the following per kg of diets: KCl, 200 mg; KI (1%), 60 mg; CoCl_2_·6H_2_O (1%), 50 mg; CuSO_4_·5H_2_O, 30 mg; FeSO_4_·H_2_O, 400 mg; ZnSO_4_·H_2_O, 400 mg; MnSO_4_·H_2_O, 150 mg; Na_2_SeO_3_·5H_2_O (1%), 65 mg; MgSO_4_·H_2_O, 2000 mg; zeolite power 3, 645.85 mg; VB1, 12 mg; riboflavin, 12 mg; VB6, 8 mg; VB12, 0.05 mg; VK3, 8 mg; inositol, 100 mg; pantothenic acid, 40 mg; niacin acid, 50 mg; folic acid, 5 mg; biotin, 0.8 mg; VA, 25 mg; VD, 35 mg; VE, 50 mg; VC, 100 mg; ethoxyquin, 150 mg; wheat flour 2, 434.15 mg.

^c^The nutritional level is the measured value.

**Table 2:** Amino acids compositions and content of HFM and fish meal (%, dry matter).

Amino acids	Hydrolyzed feather meal	Fish meal
Essential amino acids		
Methionine	0.75	1.63
Lysine	2.16	4.25
Histidine	0.88	1.78
Threonine	3.97	2.68
Arginine	5.75	3.65
Valine	5.76	3.08
Phenylalanine	3.95	2.47
Isoleucine	3.79	2.49
Leucine	6.75	4.78
Nonessential amino acids		
Alanine	4.18	4.08
Tryptophan	0.80	0.78
Proline	11.80	3.44
Cystine	4.16	0.54
Serine	8.80	2.52
Glutamic acid	4.81	8.27
Aspartic acid	2.92	5.58
Tyrosine	2.04	1.64
Glycine	8.95	4.54

**Table 3:** Amino acids compositions and content of experimental feed (%, dry matter, *n* = 3).

Items	Diets					Pr > *F*	
	FM	HFM2	HFM4	HFM6	HFM8	Linear	Quadratic
Essential amino acids							
Methionine	0.90 ± 0.11	0.87 ± 0.09	0.84 ± 0.12	0.82 ± 0.04	0.79 ± 0.07	0.39	0.99
Lysine	2.40 ± 0.05	2.34 ± 0.11	2.28 ± 0.06	2.22 ± 0.05	2.22 ± 0.19	0.18	0.79
Histidine	1.15 ± 0.05	1.13 ± 0.08	1.10 ± 0.04	1.07 ± 0.10	1.05 ± 0.06	0.29	1.00
Threonine	1.73 ± 0.02	1.74 ± 0.04	1.76 ± 0.01	1.77 ± 0.09	1.78 ± 0.11	0.51	0.99
Arginine	2.56 ± 0.09	2.59 ± 0.06	2.61 ± 0.06	2.64 ± 0.07	2.67 ± 0.05	0.24	0.99
Valine	2.14 ± 0.08	2.18 ± 0.11	2.22 ± 0.12	2.26 ± 0.12	2.30 ± 0.04	0.22	1.00
Phenylalanine	1.99 ± 0.04	2.01 ± 0.07	2.03 ± 0.07	2.05 ± 0.03	2.07 ± 0.01	0.24	0.98
Isoleucine	1.79 ± 0.12	1.80 ± 0.07	1.82 ± 0.03	1.83 ± 0.06	1.85 ± 0.11	0.59	1.00
Leucine	3.46 ± 0.13	3.48 ± 0.00	3.49 ± 0.13	3.51 ± 0.07	3.53 ± 0.02	0.51	0.99
Nonessential amino acids							
Alanine	2.34 ± 0.03	2.33 ± 0.07	2.31 ± 0.02	2.29 ± 0.01	2.28 ± 0.12	0.46	1.00
Tryptophan	0.50 ± 0.04	0.50 ± 0.02	0.50 ± 0.01	0.50 ± 0.01	0.49 ± 0.01	0.62	0.99
Proline	2.61 ± 0.01	2.77 ± 0.06	2.93 ± 0.08	3.08 ± 0.38	3.24 ± 0.18	0.03	1.00
Cystine	0.59 ± 0.08	0.66 ± 0.04	0.73 ± 0.09	0.8 ± 0.16	0.88 ± 0.04	0.03	1.00
Serine	2.03 ± 0.01	2.14 ± 0.01	2.26 ± 0.10	2.38 ± 0.10	2.50 ± 0.02	0.00	1.00
Glutamic acid	7.07 ± 0.20	6.96 ± 0.07	6.85 ± 0.08	6.74 ± 0.09	6.64 ± 0.06	0.01	0.99
Aspartic acid	3.84 ± 0.01	3.76 ± 0.01	3.68 ± 0.17	3.61 ± 0.03	3.53 ± 0.02	0.01	1.00
Tyrosine	1.33 ± 0.07	1.33 ± 0.07	1.33 ± 0.02	1.33 ± 0.05	1.33 ± 0.00	0.95	1.00
Glycine	2.29 ± 0.22	2.36 ± 0.14	2.43 ± 0.03	2.50 ± 0.14	2.57 ± 0.01	0.13	0.99
TAA	40.65 ± 1.88	40.95 ± 1.55	41.19 ± 1.50	41.41 ± 1.00	41.71 ± 0.66	0.57	1.00

Abbreviation: TAA, total amino acid.

**Table 4:** Growth performance responding to graded levels of HFM inclusion in *Pelteobagrus fulvidraco* (*n* = 3).

Items	Diets					Pr > *F*	
	FM	HFM2	HFM4	HFM6	HFM8	Linear	Quadratic
Initial body weight (IBW, g)	10.52 ± 0.24	10.48 ± 0.12	10.51 ± 0.26	10.54 ± 0.33	10.51 ± 0.14	0.97	0.98
Final body weight (FBW, g)	53.08 ± 1.39^b^	51.89 ± 0.01^ab^	51.41 ± 1.09^ab^	49.68 ± 1.02^ab^	48.46 ± 1.26^a^	0.01	0.75
Specific growth rate (SGR, %/day)^1^	2.89 ± 0.06^b^	2.86 ± 0.01^b^	2.80 ± 0.03^ab^	2.75 ± 0.06^ab^	2.73 ± 0.02^a^	0.01	0.81
Weight gain rate (WGR, %)^2^	402.50 ± 8.90^b^	395.20 ± 1.50^b^	389.35 ± 10.71^ab^	370.25 ± 12.50^ab^	363.26 ± 9.74^a^	0.01	0.73
Survival rate (SR, %)^3^	100.00 ± 0.00	99.33 ± 0.67	100.00 ± 0.00	100.00 ± 0.00	100.00 ± 0.00	0.50	0.56
Feed conversion rate (FCR)^4^	1.59 ± 0.31	1.63 ± 0.29	1.69 ± 0.22	1.73 ± 0.05	1.78 ± 0.09	0.51	0.99
Hepatosomatic index (HSI, %)^5^	1.45 ± 0.10^a^	2.24 ± 0.08^b^	2.27 ± 0.10^b^	2.53 ± 0.07^b^	2.22 ± 0.25^b^	0.00	0.00
Relative intestinal length (RIL, %)^6^	87.98 ± 6.94^a^	111.01 ± 6.18^b^	102.86 ± 4.83^ab^	97.33 ± 4.95^ab^	100.46 ± 5.23^ab^	0.54	0.11
Intestosomatic index (ISI, %)^7^	0.97 ± 0.08^a^	1.48 ± 0.1^c^	1.28 ± 0.07^bc^	1.41 ± 0.09^c^	1.10 ± 0.09^ab^	0.52	0.00
Moisture (%)	70.75 ± 0.01	71.11 ± 0.01	71.76 ± 0.02	71.82 ± 0.01	72.25 ± 0.00	0.96	0.76
Crude fat (%)	32.98 ± 0.18	32.89 ± 0.24	33.17 ± 0.23	33.62 ± 0.21	33.29 ± 0.36	0.12	0.75
Crude ash (%)	12.37 ± 0.12^ab^	12.09 ± 0.10^ab^	12.61 ± 0.18^b^	12.01 ± 0.24^ab^	11.88 ± 0.26^a^	0.11	0.28
Crude protein (%)	53.74 ± 0.42^ab^	54.51 ± 0.24^b^	53.43 ± 0.25^a^	52.47 ± 0.26^a^	54.03 ± 0.25^b^	0.64	0.53

*Note:* Values are means ± S.D. of three replicate groups. Values with different superscript letters in the same row are significantly different (*p* < 0.05).

^1^SGR = (Ln FBW − Ln IBW)/feed duration.

^2^WGR = 100 × (FBW − IBW)/IBW.

^3^SR = 100 × The final quantity of fish/the initial quantity of fish.

^4^FCR = 100 × (The amount of feed consumed/weight gain).

^5^HSI = 100 × The weight of liver/the weight of body.

^6^RIL = [The length of intestine (cm)/the length of body (cm)] × 100.

^7^ISI = [The weight of intestine (g)/the weight of body (g)] × 100.

**Table 5:** Intestinal digestive and absorptive enzymes activities responding to graded levels of HFM inclusion in *Pelteobagrus fulvidraco* (*n* = 3).

Items	Diets					Pr > *F*	
	FM	HFM2	HFM4	HFM6	HFM8	Linear	Quadratic
Amylase (U/mg protein)	0.41 ± 0.04^a^	0.46 ± 0.03^ab^	0.43 ± 0.04^ab^	0.57 ± 0.04^b^	0.56 ± 0.06^b^	0.01	0.71
Lipase (U/g protein)	28.62 ± 5.47^ab^	28.33 ± 3.41^ab^	27.94 ± 1.24^ab^	37.21 ± 4.23^b^	22.66 ± 2.93^a^	0.75	0.19
Trypsin (U/mg protein)	622.15 ± 23.99^a^	594.42 ± 53.50^a^	920.73 ± 115.07^b^	985.61 ± 101.07^b^	420.01 ± 22.41^a^	0.97	0.00
AKP (U/g protein)	2.71 ± 0.14^c^	2.12 ± 0.12^bc^	1.95 ± 0.38^ab^	1.72 ± 0.18^ab^	1.37 ± 0.18^a^	0.00	0.62
Na^+^-K^+^-ATPase (µmolPi/mgprot/h)	0.55 ± 0.02^a^	0.80 ± 0.07^ab^	0.65 ± 0.13^ab^	0.90 ± 0.05^b^	0.64 ± 0.06^a^	0.27	0.06

*Note:* Values with different superscript letters in the same row are significantly different (*p* < 0.05).

Abbreviations: AKP, alkaline phosphatase; Na^+^–K^+^–ATPase, sodium–potassium adenosine triphosphatase.

**Table 6:** Morphology of anterior intestine responding to graded levels of HFM inclusion in *Pelteobagrus fulvidraco* (*n* = 3).

Items	Diets					Pr > *F*	
	FM	HFM2	HFM4	HFM6	HFM8	Linear	Quadratic
Villus height (VH, μm)	542.07 ± 19.82^a^	600.04 ± 27.04^ab^	608.33 ± 19.33^b^	685.07 ± 20.63^c^	769.14 ± 19.57^d^	0.00	0.28
Muscle layer thickness (MT, μm)	72.97 ± 2.39^a^	81.67 ± 2.76^a^	101.72 ± 3.73^b^	117.94 ± 5.77^c^	128.57 ± 8^c^	0.00	0.74
Goblet cells number (cells/roots)	26.90 ± 2.83^a^	29.80 ± 2.13^ab^	30.10 ± 1.61^ab^	32.60 ± 2.45^ab^	35.7 ± 2.39^b^	0.01	0.94

*Note:* Values with different superscript letters in the same row are significantly different (*p* < 0.05).

**Table 7:** Serum biochemical and immune indexes and immune responding to graded levels of HFM inclusion in *Pelteobagrus fulvidraco* (*n* = 3).

Items	Diets					Pr > *F*	
	FM	HFM2	HFM4	HFM6	HFM8	Linear	Quadratic
AST (U/L)	25.04 ± 0.55^a^	27.98 ± 0.81^b^	28.28 ± 0.47^b^	30.53 ± 0.83^c^	29.97 ± 0.43^bc^	0.00	0.04
ALT (U/L)	2.73 ± 0.50	2.26 ± 0.16	2.60 ± 0.33	2.57 ± 0.21	3.22 ± 0.28	0.23	0.15
TG (mmol/L)	1.28 ± 0.02^a^	1.31 ± 0.04^a^	1.60 ± 0.05^b^	1.71 ± 0.03^b^	1.65 ± 0.04^b^	0.00	0.03
TC (mmol/L)	2.74 ± 0.05	2.79 ± 0.04	2.90 ± 0.17	2.83 ± 0.10	3.01 ± 0.09	0.11	0.85
GLU (mmol/L)	4.71 ± 0.30^a^	6.84 ± 0.19^bc^	7.83 ± 0.49^c^	6.76 ± 0.29^bc^	6.13 ± 0.51^b^	0.04	0.00
TP × 10^−3^ (mg/mL)	30.80 ± 0.75^a^	33.35 ± 1.28^bc^	35.09 ± 0.35^c^	32.35 ± 0.75^b^	33.83 ± 0.21^bc^	0.06	0.06
ACP (U/100 mL)	26.38 ± 1.49^c^	24.64 ± 0.55^bc^	21.92 ± 0.28^a^	22.49 ± 0.43^ab^	21.52 ± 0.44^a^	0.00	0.13
AKP (U/100 mL)	4.16 ± 0.07^c^	3.38 ± 0.03^a^	3.30 ± 0.03^a^	3.83 ± 0.13^b^	3.26 ± 0.07^a^	0.00	0.01
IgM (g/L)	0.35 ± 0.04^b^	0.33 ± 0.01^b^	0.29 ± 0.01^b^	0.24 ± 0.01^a^	0.23 ± 0.01^a^	0.00	0.86
C3 (g/L)	0.13 ± 0.01^b^	0.12 ± 0.01^ab^	0.10 ± 0.00^a^	0.10 ± 0.00^a^	0.10 ± 0.00^a^	0.01	0.11
C4 (g/L)	0.21 ± 0.02^c^	0.17 ± 0.00^b^	0.16 ± 0.00^ab^	0.13 ± 0.00^a^	0.13 ± 0.01^a^	0.00	0.06

*Note:* Values with different superscript letters in the same row are significantly different (*p* < 0.05).

Abbreviations: ACP, acid phosphatase; AKP, alkaline phosphatase; ALT, alanine aminotransferase; AST, aspartate aminotransferase; C3, Complement 3; C4, Complement 4; Glu, glucose; IgM, immune globulin M; TC, total cholesterol; TG, total triglycerides; TP, total protein.

**Table 8:** Muscle physicochemical index responding to graded levels of HFM inclusion of *Pelteobagrus fulvidraco* (*n* = 3).

Items	Diets					Pr > *F*	
	FM	HFM2	HFM4	HFM6	HFM8	Linear	Quadratic
ODM (ng/g)^1^	145.82 ± 4.42^a^	147 ± 1.11^a^	150.25 ± 2.48^a^	169.53 ± 5.04^b^	155.91 ± 4.25^a^	0.01	0.35
Centrifugal loss (%)^2^	21.00 ± 4.36	22.55 ± 0.70	23.13 ± 4.65	21.02 ± 5.38	24.97 ± 1.21	0.61	0.88
Cooking loss (%)^3^	33.96 ± 1.75	32.05 ± 0.14	30.47 ± 4.00	32.33 ± 1.06	29.69 ± 2.66	0.3	0.83
Hardness (*N*)	5.77 ± 0.09^a^	5.72 ± 0.27^a^	6.38 ± 0.13^b^	6.69 ± 0.22^b^	6.29 ± 0.10^ab^	0.01	0.14
Cohesiveness	0.58 ± 0.04	0.61 ± 0.02	0.59 ± 0.01	0.59 ± 0.01	0.59 ± 0.04	0.98	0.71
Springiness (mm)	1.58 ± 0.06^b^	1.11 ± 0.22^a^	1.29 ± 0.13^ab^	1.47 ± 0.13^ab^	1.47 ± 0.07^ab^	0.74	0.09
Gumminess	3.59 ± 0.25	3.73 ± 0.51	3.20 ± 0.31	3.59 ± 0.31	3.64 ± 0.33	0.98	0.59
Chewiness (mJ)	5.59 ± 0.47	6.51 ± 0.87	4.28 ± 0.39	5.59 ± 0.86	4.75 ± 0.51	0.24	1.00
Adhesiveness	0.03 ± 0.00^a^	0.04 ± 0.00^ab^	0.04 ± 0.01^b^	0.05 ± 0.00^b^	0.04 ± 0.00^ab^	0.02	0.03

*Note:* Values with different superscript letters in the same row are significantly different (*p* < 0.05).

^1^Optical density of muscle.

^2^Centrifugal loss (%) = 100 × [(The weight of the muscle before centrifugation − the weight of the muscle after centrifugation)/the weight of the muscle before centrifugation].

^3^Cooking loss (%) = 100 × [(The weight of muscle before cooking − the weight of muscle after cooking)/the weight of muscle before cooking].

**Table 9:** Effect of HFM instead of fish meal on free amino acid content in muscle of *Pelteobagrus fulvidraco* (×10^−2^ mg/g, *n* = 3).

Items	Diets					Pr > *F*	
	FM	HFM2	HFM4	HFM6	HFM8	Linear	Quadratic
Essential amino acids							
Histidine	8.11 ± 0.25^a^	7.39 ± 0.28^a^	9.15 ± 0.33^b^	7.7 ± 0.25^a^	7.66 ± 0.16^a^	0.48	0.09
Threonine	6.93 ± 0.25	6.11 ± 0.33	5.79 ± 0.21	6.92 ± 1.38	7.6 ± 0.17	0.33	0.10
Methionine	2.16 ± 0.04	2.13 ± 0.04	2.01 ± 0.05	1.91 ± 0.14	2.11 ± 0.05	0.22	0.12
Arginine	2.45 ± 0.12	2.43 ± 0.71	2.09 ± 0.13	2.67 ± 0.78	2.13 ± 0.07	0.98	0.95
Valine	3.99 ± 0.04	3.79 ± 0.68	3.34 ± 0.11	3.71 ± 0.37	4.33 ± 0.11	0.61	0.09
Isoleucine	2.36 ± 0.01^ab^	2.11 ± 0.38^ab^	1.99 ± 0.07^a^	2.35 ± 0.14^ab^	2.63 ± 0.01^b^	0.21	0.05
Leucine	6.17 ± 0.24^ab^	5.32 ± 0.49^a^	5.3 ± 0.09^a^	6.09 ± 0.4^ab^	6.39 ± 0.04^b^	0.24	0.02
Phenylalanine	2.69 ± 0.10	2.69 ± 0.10	2.69 ± 0.10	2.69 ± 0.10	2.69 ± 0.10	0.98	0.98
Lysine	13.83 ± 0.41	11.54 ± 2.2	10.33 ± 0.34	15.39 ± 2.83	11.1 ± 0.24	0.76	0.72
Nonessential amino acids							
Glycine^1^	42.19 ± 1.38^a^	46.66 ± 6.02^ab^	57.81 ± 1.91^cd^	53.35 ± 0.38^bc^	64.75 ± 1.47^d^	0.00	0.88
Aspartic acid^1^	2.34 ± 0.45	2.84 ± 0.07	2.41 ± 0.62	2.16 ± 0.12	2.62 ± 0.01	0.92	0.94
Glutamic acid^1^	6.21 ± 0.18^a^	8.26 ± 0.81^b^	8.56 ± 0.19^bc^	8.74 ± 0.99^bc^	10.27 ± 0.24^c^	0.00	0.62
Serine	6.90 ± 0.41^a^	7.71 ± 0.05^ab^	8.13 ± 0.21^b^	8.30 ± 0.44^b^	9.65 ± 0.15^c^	0.00	0.47
Alanine^1^	17.18 ± 0.6^a^	17.78 ± 1.89^a^	19.93 ± 0.66^a^	18.67 ± 0.38^a^	24.74 ± 0.58^b^	0.00	0.07
Proline	2.31 ± 0.05	2.62 ± 0.87	2.08 ± 0.08	2.88 ± 0.94	3.70 ± 0.12	0.13	0.30
Cysteine	0.53 ± 0.02	0.59 ± 0.20	0.85 ± 0.00	0.52 ± 0.13	0.51 ± 0.08	0.76	0.11
Tyrosine	2.37 ± 0.10^a^	2.22 ± 0.09^a^	2.3 ± 0.06^a^	2.18 ± 0.13^a^	2.72 ± 0.04^b^	0.04	0.01
Tryptophan	0.64 ± 0.03^ab^	0.56 ± 0.03^a^	0.72 ± 0.02^bc^	0.60 ± 0.04^a^	0.75 ± 0.03^c^	0.03	0.12
TAA	129.36 ± 4.54^a^	132.59 ± 2.88^a^	145.14 ± 3.96^a^	146.42 ± 10.13^a^	166.52 ± 3.45^b^	0.00	0.31

*Note:* Values with different superscript letters in the same row are significantly different (*p* < 0.05).

Abbreviation: TAA, total amino acid.

^1^Flavor amino acids.

**Table 10:** Skin pigments of *Pelteobagrus fulvidraco* fed the experiment diets with gradient levels of HFM (*n* = 3).

Position	Items	Diets					Pr > *F*	
		FM	HFM2	HFM4	HFM6	HFM8	Linear	Quadratic
Dorsal skin	Tyrosinase (ng/g)	10.59 ± 0.69^b^	9.28 ± 0.44^ab^	8.53 ± 0.14^a^	8.98 ± 0.26^a^	8.57 ± 0.40^a^	0.01	0.09

Ventral skin	Lutein (ug/g)	5.33 ± 0.09^b^	5.08 ± 0.14^b^	4.70 ± 0.12^a^	4.79 ± 0.04^a^	4.79 ± 0.08^a^	0.00	0.01
	Carotene (ng/g)	45.12 ± 1.78^b^	42.99 ± 1.95^ab^	41.31 ± 0.25^ab^	41.90 ± 0.07^ab^	39.86 ± 0.50^a^	0.01	0.60

*Note:* Values with different superscript letters in the same row are significantly different (*p* < 0.05).

## Data Availability

The data will be made available upon request.
